# Research hotspots and emerging trends of deep learning applications in orthopedics: A bibliometric and visualized study

**DOI:** 10.3389/fpubh.2022.949366

**Published:** 2022-07-19

**Authors:** Chengyao Feng, Xiaowen Zhou, Hua Wang, Yu He, Zhihong Li, Chao Tu

**Affiliations:** ^1^The Department of Orthopaedics, The Second Xiangya Hospital of Central South University, Changsha, China; ^2^Hunan Key Laboratory of Tumor Models and Individualized Medicine, The Second Xiangya Hospital of Central South University, Changsha, China; ^3^Xiangya School of Medicine, Central South University, Changsha, China; ^4^The Department of Radiology, The Second Xiangya Hospital of Central South University, Changsha, China

**Keywords:** orthopedics, deep learning, bibliometric analysis, research trends, Citespace

## Abstract

**Background:**

As a research hotspot, deep learning has been continuously combined with various research fields in medicine. Recently, there is a growing amount of deep learning-based researches in orthopedics. This bibliometric analysis aimed to identify the hotspots of deep learning applications in orthopedics in recent years and infer future research trends.

**Methods:**

We screened global publication on deep learning applications in orthopedics by accessing the Web of Science Core Collection. The articles and reviews were collected without language and time restrictions. Citespace was applied to conduct the bibliometric analysis of the publications.

**Results:**

A total of 822 articles and reviews were finally retrieved. The analysis showed that the application of deep learning in orthopedics has great prospects for development based on the annual publications. The most prolific country is the USA, followed by China. University of California San Francisco, and Skeletal Radiology are the most prolific institution and journal, respectively. LeCun Y is the most frequently cited author, and *Nature* has the highest impact factor in the cited journals. The current hot keywords are convolutional neural network, classification, segmentation, diagnosis, image, fracture, and osteoarthritis. The burst keywords are risk factor, identification, localization, and surgery. The timeline viewer showed two recent research directions for bone tumors and osteoporosis.

**Conclusion:**

Publications on deep learning applications in orthopedics have increased in recent years, with the USA being the most prolific. The current research mainly focused on classifying, diagnosing and risk predicting in osteoarthritis and fractures from medical images. Future research directions may put emphasis on reducing intraoperative risk, predicting the occurrence of postoperative complications, screening for osteoporosis, and identification and classification of bone tumors from conventional imaging.

## Introduction

As a subset of machine learning, deep learning has broken the limitations of traditional machine learning, and can implement more accurate classification and segmentation of images to extract feature elements (1, 2). It is known that deep learning surpasses the performance of other machine learning techniques in several aspects, such as predicting the potential activity of drug molecules (3) and the alternative splicing patterns in tissues (4). Convolutional neural networks (CNNs), consisting of multiple neural layers, are powerful learning structures in deep learning. They are effectively used to solve related problems in computer vision and images. By varying the depth and breadth of the neural layers, the capacity of the CNNs can be expanded to accommodate tens of thousands of images and allow them to interpret correctly (1). Currently, deep learning has been demonstrated to have the potential to identify diseases such as skin lesions and diabetic retinopathy, and it has achieved greater accuracy that comparable to experienced physicians (5, 6).

Most musculoskeletal diseases in orthopedics require a large degree of help from images. Given the advantages of deep learning in image processing, there has been a growing number of researches on the application of deep learning in orthopedics in recent years (7). Bibliometrics is a statistical and quantitative method to analyze the academic characteristics of a body of literatures in certain scientific fields. It can help researchers to grasp the priorities and trends, and predict its prospects. Therefore, it has been applied in multiple research areas, including immunology (8), oncology (9, 10), nursing (11), vaccine (12), and orthopedics (13). The software Citespace can quantitatively analyze the literature database to evaluate the distribution of countries (or regions) and institutions, as well as excavate the literature that plays a central position in the research field, and visualize the research hotspots and development trends (14).

Currently, no bibliometric analysis has been conducted to quantitatively analyze the progress and current status of deep learning in this emerging field. Herein, this study aimed to elucidate the research hotspots, key fields, and trends of deep learning applications in orthopedics in recent years by using Citespace, Moreover, the research direction and references for further exploration were shown as well.

## Methods

### Data source and collection

Since the details of the documents in the Web of Science are more accurate than other databases, such as Scopus, PubMed, Embase, etc. We retrieved all literature from the Web of Science Core Collection. The retrieval was completed within 1 day on March 29, 2022 to reduce changes due to frequent updates to the bibliographic database. The searching strategy was as follows: #1 and #2 (#1:WC = (orthopedics) OR TS = (orthopedic^*^ OR orthopedic^*^ OR “sports medicine”) OR TS = (“bone disease^*^” OR TS = “bone age” OR bone fracture^*^ OR “joint disease^*^” OR “joint disorder^*^” OR TS = “arthritis” OR TS = “joint dislocations” OR TS = “joint dislocations” OR TS = “musculoskeletal system” OR “musculoskeletal disease^*^” OR “musculoskeletal disorder^*^” OR “musculoskeletal trauma^*^” OR TS = “bone tumor^*^” OR TS = “bone cancer” OR TS = “bone metastasis” OR TS = “bone cyst^*^” OR TS = “soft tissue tumor^*^” OR TS = “soft tissue neoplasm^*^” OR TS = “joint replacement^*^” OR TS = “arthroplasty” OR TS = “arthroscope^*^”), #2: TS =“deep learning” OR TS = “convolutional neural network^*^”). There is no limit to the publication year. The type of literature was selected as articles and reviews without language restriction. Literature not relevant to this topic was excluded, and duplicate literature was removed by Using Citespace (5.8 R3). Finally, a total of 822 articles were retrieved and exported for records in the format of plain text files. Meanwhile, we also obtained the number of annual publications and the amount of publication of the journal.

### Statistical analysis and visualization

The number of annual publications was imported into Microsoft Office Excel 2021 and the trend was further analyzed. CiteSpace is a visual bibliometrics tool based on Java language for quantitative analysis of literature in specific research areas. The conception of “co-citation analysis theory” is as follows: when two documents appeared in the reference list of a third cited document, these two documents form a co-citation relationship. By mining the “co-introduction relationship”, it is possible to reveal important turning points, the evolution of keywords, and the frontiers in related research fields. In this study, the time spans were set to 2015 to 2022 based on the publication date of all 822 literature. Time slices were set to 1 year per slice, and selection criteria was selected as g-index. Authors, countries, and institutions were selected to perform the cooperative network analysis. Keywords were selected for co-occurrence, burst, cluster analysis, and timeline viewer. Reference, cited-authors, and cited journals were chosen for citation analysis. Cosine was select for the link strength. Besides, pathfinder, pruning sliced networks, and pruning the merged network in pruning algorithm were selected. In the visual network maps, node colors change from cold to warm from the inside to the outside, representing the year from the original to the most recent. The purple circle on the outside represented high centrality, and the node connection indicated cooperation, co-occurrence, and co-citation.

## Results

### Trends of annual publications

**Figure 1:**
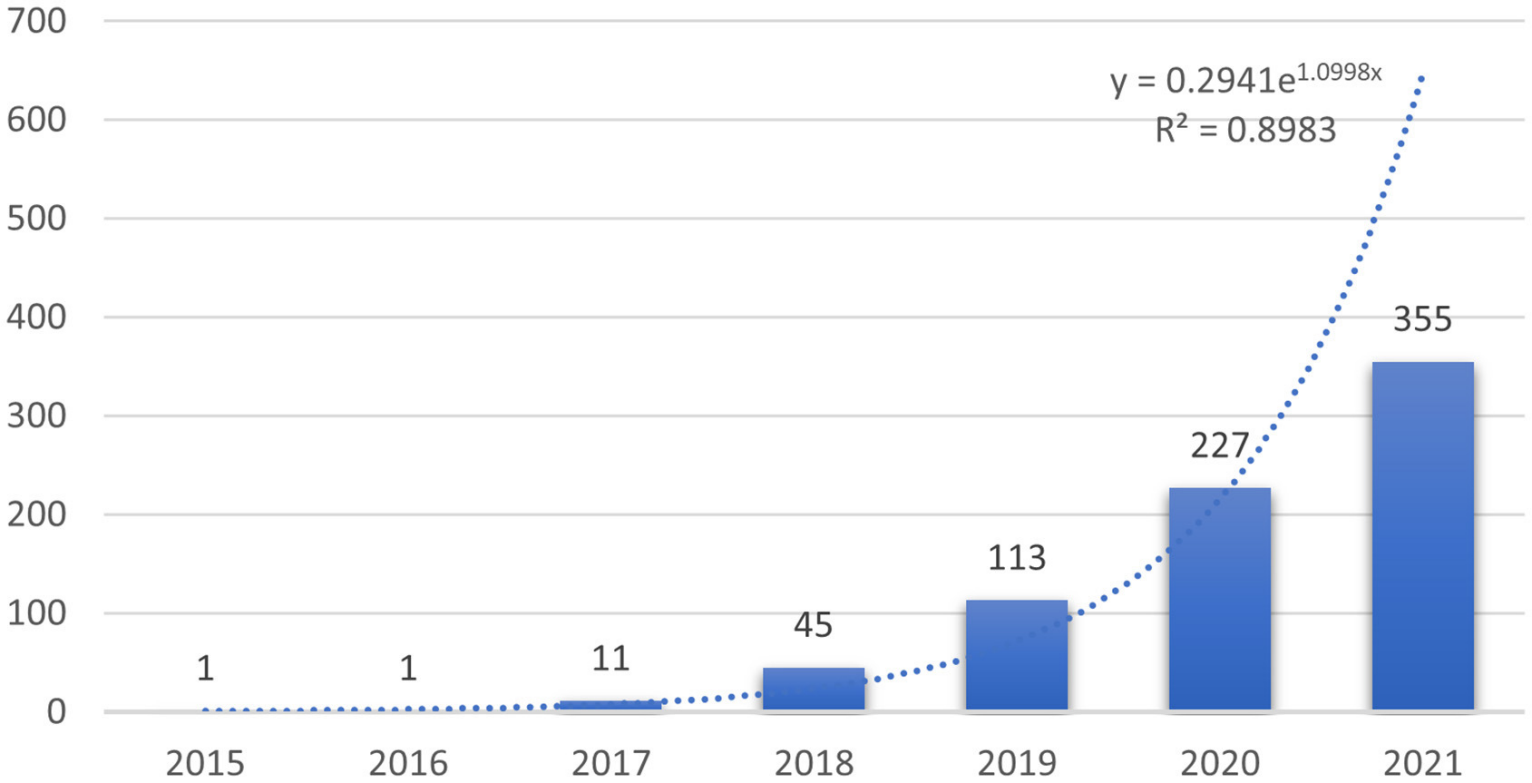
Number and trend of annual publications.

Overall, a total of 822 publications (759 articles and 63 reviews) were identified. The number of studies published in each year can help us understand the general trends in the relevant research. Since 2022 has just begun, the number of documents in 2022 cannot show the overall publication situation. Therefore, the analysis only included the publication from 2015 to 2021. As shown in Figure 1, the number of studies concerning the application of deep learning in orthopedics has increased year by year since 2017, indicating that deep learning has received high attention from orthopedics in this period. Compared with publications in 2019, the total number of articles published in 2020 were 227, showing an explosive growth. To further understand the trend of annual publications in this field between 2015 and 2021, a trend line of publications in this period was plotted, and the results showed the exponential function Y = 0.2941e1.0998x (R2 = 0.8983, Y is the annual publication, and X is the year). It is clear that the application of deep learning in orthopedics has great potential, and research trends are likely to continue.

### Distribution of countries/ regions and institutions

**Figure 2:**
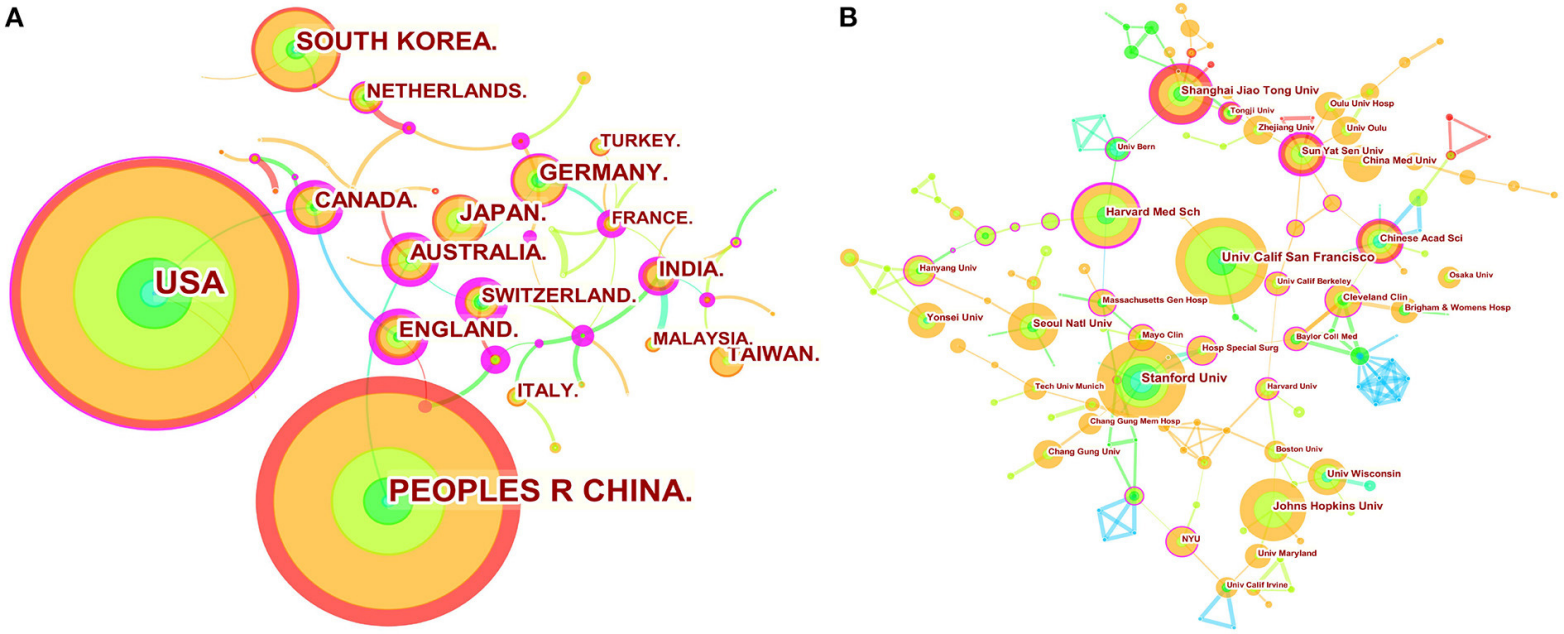
Map of countries (or regions) cooperation networks (**A)** and institution cooperation networks **(B)**. The nodes represent country (or region) or institution. The lines represent cooperation relationships. The colors in the nodes represent the years, and the purple ring represents centrality.

We used Citespace to conduct co-citation analysis of the countries (or regions) and institutions. The country or region distribution map consisted of 56 nodes and 53 links. As demonstrated in Figure 2A and Table 1, the most significant number of publications came from USA (211 time) and China (196 time), which were three times higher than those of other countries or regions. The countries of the top ten centrality were Greece (0.76), Switzerland (0.75), Spain (0.74), France (0.51), Estonia (0.5), England (0.45), Australia (0.45), Egypt (0.45), Canada (0.42), and Belgium (0.41) (Table 2). Analyzing nodes reveals that they have more connections to other countries and continents. England, Australia, and Canada had high centrality and publications. The institution distribution map consisted of 225 nodes and 239 links. As shown in Figure 2B and Table 2, the top ten prolific institutions were University of California San Francisco (USA), Stanford University (USA), Johns Hopkins University (USA), Harvard Medical School (USA), Shanghai Jiao Tong University (China), Seoul National University (South Korea), University of Chinese Academy of Sciences (China), Sun Yat-sen University (China), China Medical University (China), and Yonsei University (South Korea). Nodes with a centrality >0.1 indicate a good key role. Sun Yat-sen University (China), Harvard Medical School (USA), Shanghai Jiao Tong University (China), and University of Chinese Academy of Sciences (China) had high centrality and publication.

**Table 1:** The top 10 countries (or regions) and institutions with the most publications.

**Rank**	**Country or regions**	**Centrality**	**Count**	**Institution**	**Centrality**	**Count**
1	USA	0.23	211	University of California San Francisco (USA)	0.03	24
2	China	0.00	196	Stanford University (USA)	0.05	23
3	South Korea	0.07	70	Johns Hopkins University (USA)	0.06	17
4	Germany	0.11	44	Harvard Medical School (USA)	0.24	17
5	Japan	0.00	44	Shanghai Jiao Tong University (China)	0.18	16
6	England	0.45	38	Seoul National University (South Korea)	0.05	13
7	Canada	0.42	37	University of Chinese Academy of Sciences (China)	0.18	12
8	Australia	0.45	36	Sun Yat-sen University (China)	0.25	11
9	India	0.25	29	China Medical University (China)	0.04	10
10	Taiwan	0.00	29	Yonsei University (South Korea)	0.00	10

**Table 2:** The top 10 countries (or regions) and institutions with the most centrality.

**Rank**	**Country or regions**	**Centrality**	**Count**	**Institution**	**Centrality**	**Count**
1	Greece	0.76	11	Sun Yat-sen University (China)	0.25	11
2	Switzerland	0.75	27	Harvard Medical School (USA)	0.24	17
3	Spain	0.74	6	University of California, Berkeley (USA)	0.19	6
4	France	0.51	18	Shanghai Jiao Tong University (China)	0.18	16
5	Estonia	0.5	3	University of Chinese Academy of Sciences (China)	0.18	12
6	England	0.45	38	University of Amsterdam (Netherlands)	0.18	4
7	Australia	0.45	36	Northwestern Polytech University (China)	0.18	4
8	Egypt	0.45	1	Tongji University (China)	0.17	6
9	Canada	0.42	37	Duke NUS Medical School (Singapore)	0.17	2
10	Belgium	0.41	7	Massachusetts General Hospital (USA)	0.16	7


### Analysis of authors and cited authors

**Figure 3:**
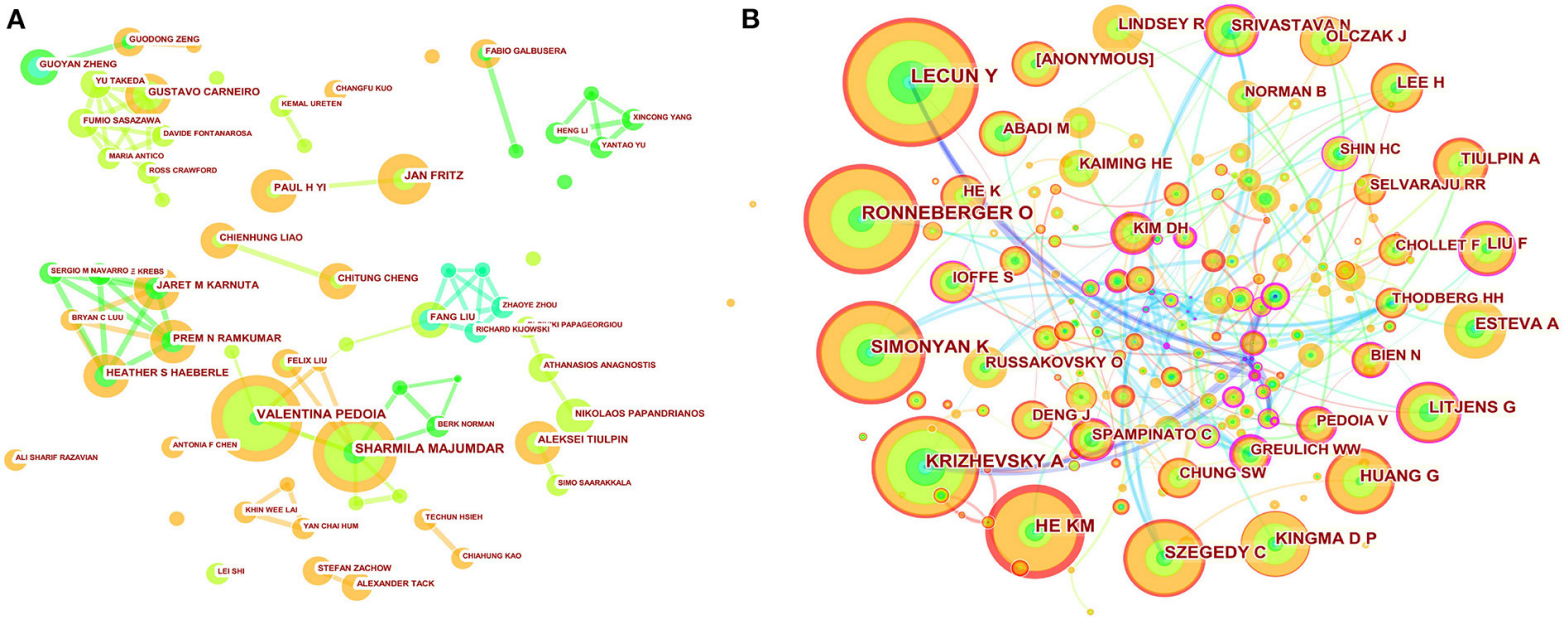
Map of author's cooperative relationship **(A)** and co-citation network **(B)**. The nodes represent author or co-cited author, and the lines represent cooperation or co-citation relationships, respectively. The colors in the nodes represent the years, and the purple ring represents centrality.

A total of 250 authors were involved in the cooperative map (Figure 3A). As shown in Table 3, VALENTINA PEDOIA was the author with the most published literature. Interestingly, among the top ten authors, we found three major collaborative networks. They were VALENTINA PEDOIA (University of California System, USA) and SHARMILA MAJUMDAR (University of California System, USA), PAUL H YI (Johns Hopkins University, USA) and JAN FRITZ (New York University, USA), JARET M KARNUTA (Cleveland Clinic Foundation, USA), PREM N RAMKUM AR (Cleveland Clinic Foundation, USA) and HEATHER S HAEBERLE (Cleveland Clinic Foundation, USA), separately. Co-cited authors are two (or more authors) who are cited in one or more subsequent papers at the same time, the two or more authors constitute a co-cited relationship. The co-cited author map consisted of 487 notes and 747 links (Figure 3B). As displayed in Table 3, LeCun Y was the most frequently co-cited author. Among the top ten co-cited authors, Litjens G had the most centrality.

**Table 3:** The top 10 authors and co-cited authors with the most counts.

**Rank**	**Author**	**Centrality**	**Count**	**Co-cited author**	**Centrality**	**Count**
1	VALENTINA PEDOIA	0.00	12	LeCun Y	0.03	166
2	SHARMILA MAJUMDAR	0.00	11	Ronneberger O	0.02	142
3	JAN FRITZ	0.00	7	Simonyan K	0.02	134
4	PREM N RAMKUMAR	0.00	6	Krizhevsky A	0.07	133
5	JARET M KARNUTA	0.00	6	He KM	0.00	123
6	PAUL H YI	0.01	6	Szegedy C	0.06	103
7	HEATHER S HAEBERLE	0.00	6	Kingma D P	0.00	87
8	GUSTAVO CARNEIRO	0.00	6	Huang G	0.00	84
9	ALEKSEI TIULPIN	0.00	6	Litjens G	0.12	78
10	GUOYAN ZHENG	0.00	5	Esteva A	0.00	75

### Journals and co-cited academic journals

**Figure 4:**
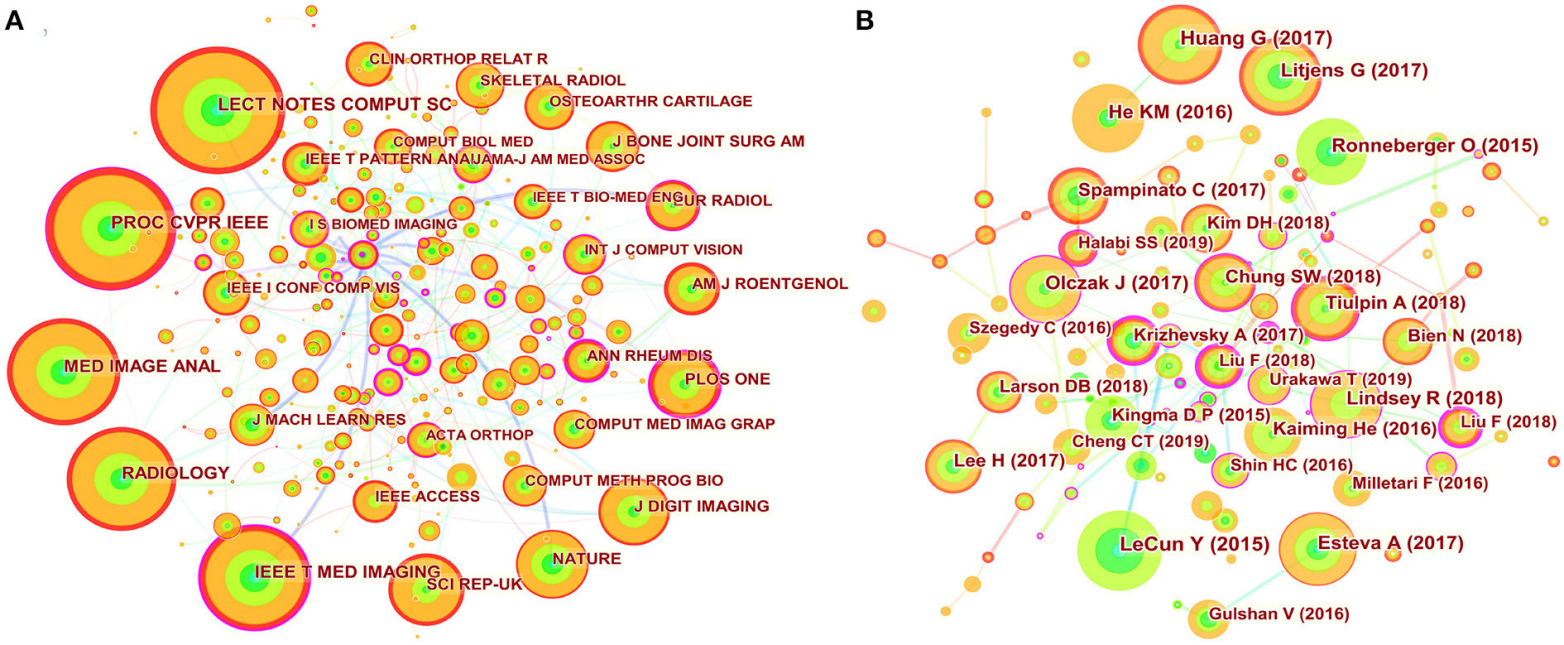
Map of journal co-citation and cited references. **(A)** The nodes represent journal. The lines represent co-citation relationships. The colors in the nodes represent the years, and the purple ring represents centrality. **(B)** The nodes represent cited reference. The lines represent co-citation relationships. The colors in the nodes represent the years, and the purple ring represents centrality.

The 822 articles retrieved in this study were published in 308 journals. As shown in Table 4, The journal *Skeletal Radiology* (33 times) had the highest number of outputs, followed by *IEEE Access* (28 times), *Scientific Reports* (25 times), *Computer Methods and Programs in Biomedicine* (19 times), *Applied Sciences Basel* (17 times), *Diagnostics* (17 times), *International Journal of Computer Assisted Radiology and Surgery* (15 times), *Journal of Digital Imaging* (14 times), *Medical Image Analysis* (14 times), and *Sensors* (14 times). The co-occurrence analysis of cited journals obtained from Citespace was shown in Figure 4A and Table 4, with 532 nodes and 817 links. The greater the node, the higher the co-citation frequency of journals. Through the analysis of cited journals, the distribution of journals that focus on this field can be obtained. Co-citation frequency reflects the quality and influence of journals. Journals with high co-citation frequency are often regarded as mainstream journals. The top ten cited journals were *Lecture Notes in Computer Science* (345 times), *In Proceedings of The IEEE Conference on Computer Vision and Pattern Recognition* (324 times), *Medical Image Analysis* (287 times), *Radiology* (280 times), *IEEE Transactions on Medical Imaging* (274 times), *Scientific Reports* (197 times), *Nature* (190 times), *Journal of Digital Imaging* (185 times), *PLoS One* (179 times), and *American Journal of Roentgenology* (149 times).

**Table 4:** The top 10 journals and cited journals with the most publications or citation.

**Rank**	**Journal**	**Publication**	**IF (2021)**	**JCR**	**Co-cited journal**	**Citation**	**IF (2021)**	**JCR**
1	*Skeletal Radiology*	33	2.199	Q3	*Lecture Notes in Computer Science*	345	Not available	
2	*IEEE Access*	28	3.367	Q2	*In Proceedings of The IEEE Conference on Computer Vision and Pattern Recognition*	324	Not available	
3	*Scientific Reports*	25	4.38	Q1	*Medical Image Analysis*	287	8.545	Q1
4	*Computer Methods and Programs in Biomedicine*	19	5.428	Q1	*Radiology*	280	11.105	Q1
5	*Applied Sciences Basel*	17	2.679	Q2	*IEEE Transactions on Medical Imaging*	274	10.048	Q1
6	*Diagnostics*	17	3.706	Q2	*Scientific Reports*	197	4.38	Q1
7	*International Journal of Computer Assisted Radiology and Surgery*	15	2.924	Q2	*Nature*	190	49.962	Q1
8	*Journal of Digital Imaging*	14	4.056	Q1	*Journal of Digital Imaging*	185	4.056	Q1
9	*Medical Image Analysis*	14	8.545	Q1	*PLoS One*	179	3.24	Q2
10	*Sensors*	14	3.576	Q1	*American Journal of Roentgenology*	149	3.959	Q2


### Analysis of cited references

A total of 464 nodes and 733 links presenting the co-citation relationship of the references formed the cited reference network in Figure 4B. Co-citation means that two or more articles are cited by one or more papers at the same time, and important references have higher co-citation frequency. As shown in Table 5, among the top ten most frequently cited references, “Deep learning” had the most frequency. Meanwhile, there were three proceedings papers, namely the “Densely Connected Convolutional Networks,” “Deep Residual Learning for Image Recognition,” and “U-Net: Convolutional Networks for Biomedical Image Segmentation.” Most of these articles were preliminary explorations of the neighborhood, and the publication of these articles was related to the gradual increase in annual publications that began in 2017.

**Table 5:** Top 10 cited references on the applications of deep learning in Orthopedics.

**Rank**	**Reference**	**Year**	**Author**	**Type of study**
1	Deep learning	2015	LeCun Y	Review
2	Densely Connected Convolutional Networks	2017	Huang G	Proceedings paper
3	A survey on deep learning in medical image analysis	2017	Litjens G	Article
4	Dermatologist-level classification of skin cancer with deep neural networks	2017	Esteva A	Article
5	Deep Residual Learning for Image Recognition	2016	He KM	Proceedings paper
6	Deep neural network improves fracture detection by clinicians	2018	Lindsey R	Article
7	Artificial intelligence for analyzing orthopedic trauma radiographs	2017	Olczak J	Article
8	U-Net: Convolutional Networks for Biomedical Image Segmentation	2015	Ronneberger O	Proceedings paper
9	Automatic Knee Osteoarthritis Diagnosis from Plain Radiographs: A Deep Learning-Based Approach	2018	Tiulpin A	Article
10	Deep learning for automated skeletal bone age assessment in X-ray images	2017	Spampinato C	Article

### Analysis of keywords, burst value, clustering, and time evolution

**Figure 5:**
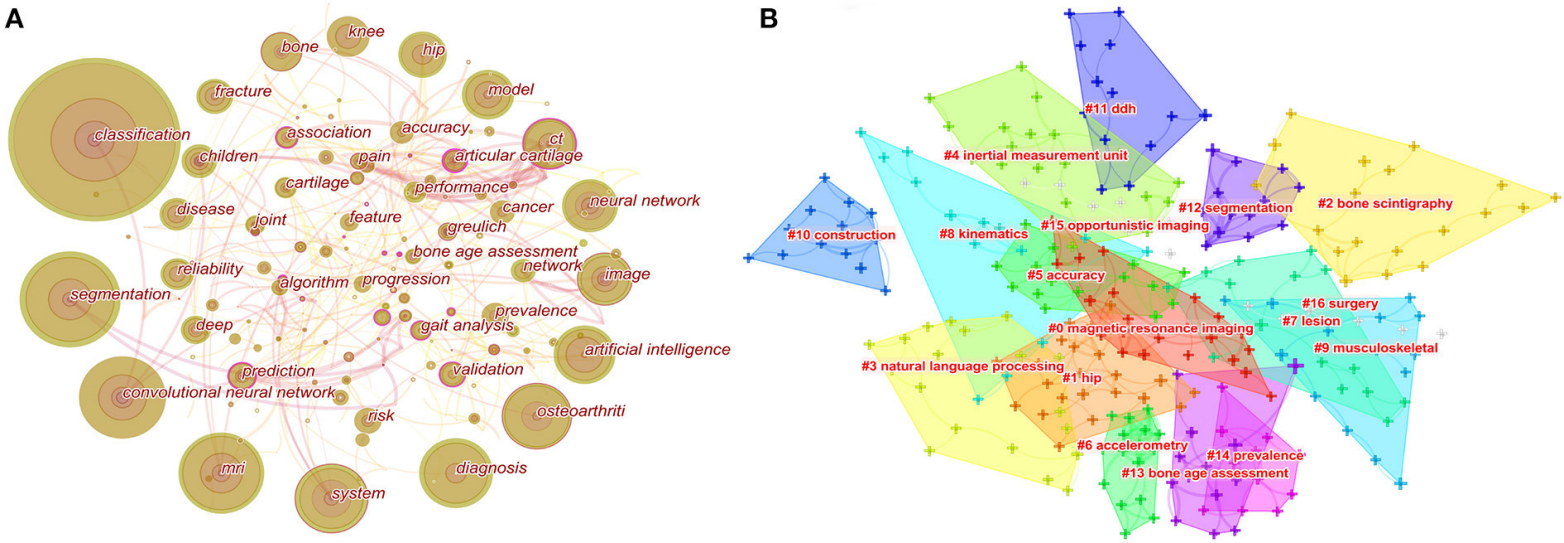
Map of keywords occurrence (**A**) and the clustering of keywords (**B**). For keywords occurrence, the nodes represent keywords. The lines represent co-occurrence relationships, and the colors in the nodes represent the years.

The high-frequency keywords the article can help us understand the main research hotspots of the filed. Keyword co-occurrence analysis was performed by Citespcace. The co-occurrence network of keywords had a total of 265 nodes and 404 links (Figure 5A). The high-frequency keywords were listed in Table 6. The top 20 keywords were as follows: classification, segmentation, convolutional neural network, MRI, system, diagnosis, osteoarthritis, artificial intelligence, image, neural network, CT, model, hip, knee, bone, fracture, children, reliability, disease, and prediction. From these keywords, we found that the main research methods were artificial intelligence, neural network, and convolutional neural network. The main research tasks were classification, segmentation, diagnosis, and prediction. The main research content was image, and the main research objects were bone, hip, knee, osteoarthritis, fracture, and disease. To sum up, classification, diagnosis, and risk prediction of various types of fractures and osteoarthritis by image were major research hot-spots.

**Table 6:** The top 20 keywords with the most citation count.

**Rank**	**Keyword**	**Frequency**	**Rank**	**Keyword**	**Frequency**
1	Classification	110	11	CT	35
2	Segmentation	65	12	Model	34
3	Convolutional neural network	56	13	Hip	32
4	MRI	56	14	Knee	28
5	System	48	15	Bone	27
6	Diagnosis	47	16	Fracture	25
7	Osteoarthritis	46	17	Children	24
8	Artificial intelligence	41	18	Reliability	22
9	Image	37	19	Disease	22
10	Neural network	37	20	Prediction	19

The clustering of keywords can present the structural system of related research fields. The sixteen different clusters made through Citespace were shown in Figure 5B: #0 magnetic resonance imaging, #1 hip, #2 bone scintigraphy, #3 natural language processing, #4 inertial measurement unit, #5 accuracy, #6 accelerometry, #7 lesion, #8 kinematics, #9 musculoskeletal, #10 construction, #11 DDH, #12 segmentation, #13 bone age assessment, #14 prevalence, #15 opportunistic imaging, and #16 surgery. The ordinal number of clusters was arranged by cluster size, and the smaller the ordinal number, the larger the cluster.

**Figure 6:**
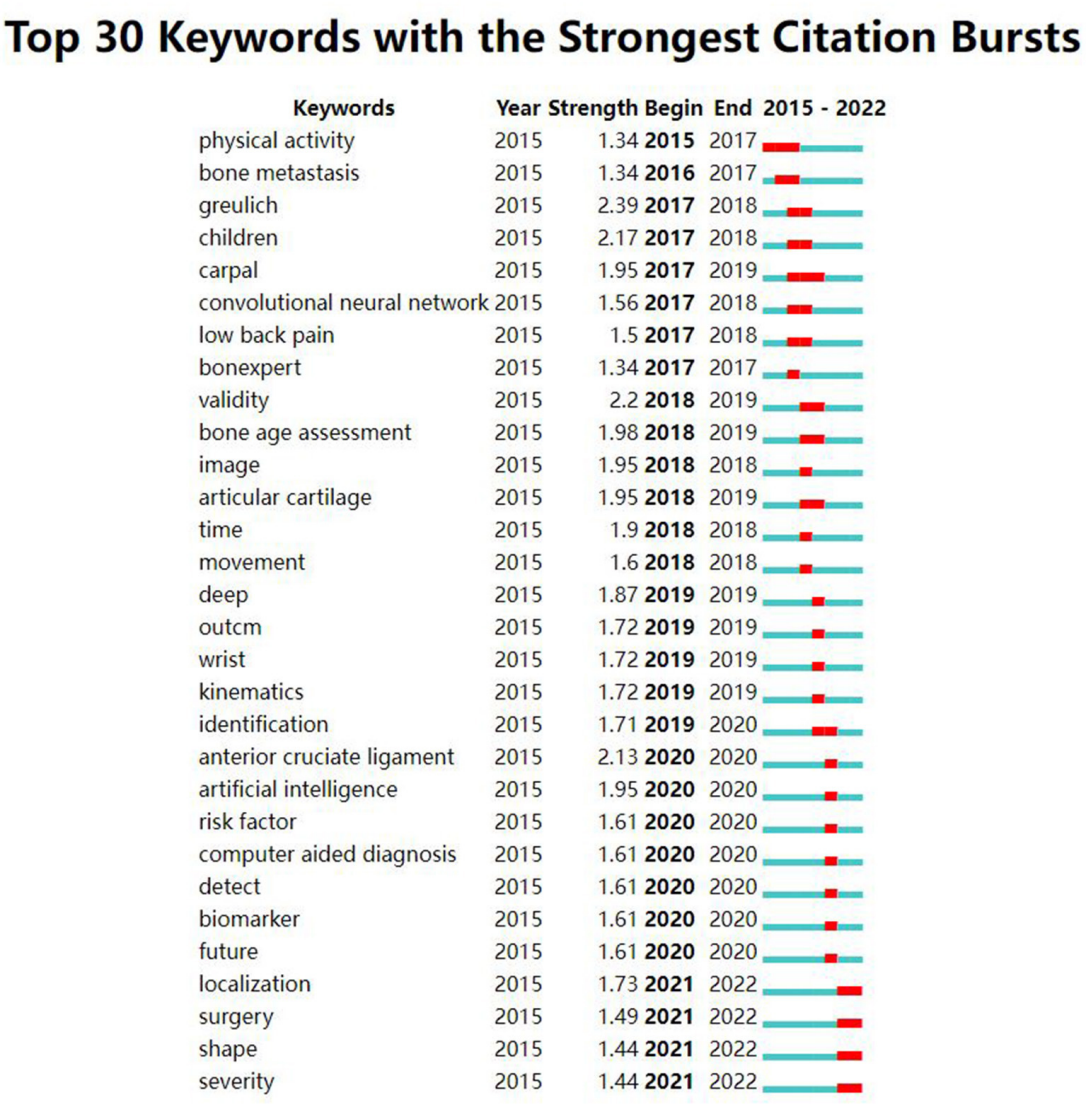
The top 30 keywords with the strongest citation bursts.

**Figure 7:**
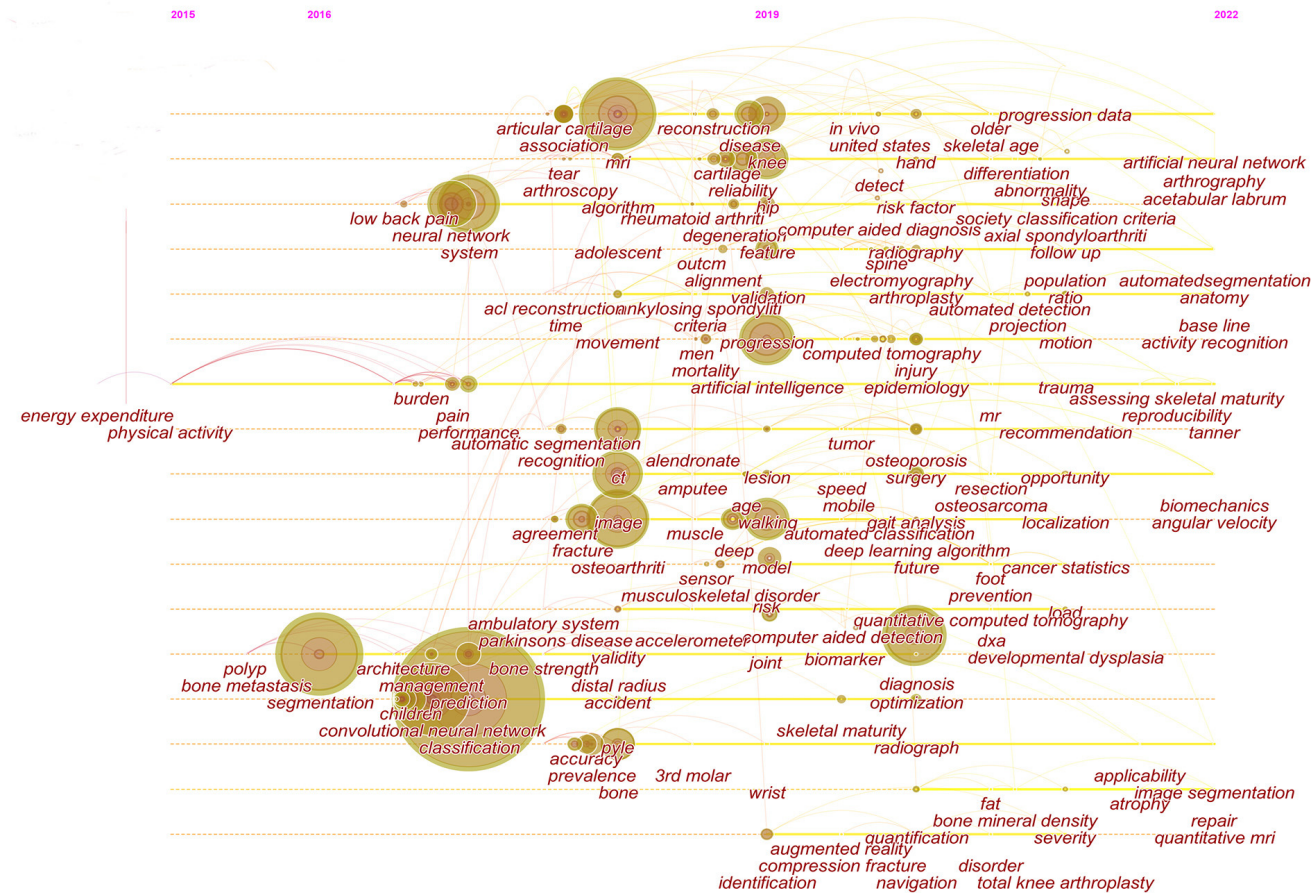
The timeline view of keywords.

The strong burst keywords can help us explore the development trends of the filed. We use Citespace's burst function to analyze keywords that have attracted much attention from academia. The burst keywords were shown in Figure 6. Keywords with higher burst values over a period mean that they have received special attention during the corresponding time intervals and may become a new research trend. In the burst keywords after 2020, we paid attention to the following main keywords: risk factor, identification, localization, and surgery. At the same time, a timeline viewer of keywords was plotted, which may help to visualize the phased hotspots and directions from the temporal dimension (Figure 7).

## Discussion

This study utilized the principles of bibliometrics analysis and the method of Citespace visualization. In the application of deep learning in orthopedic filed, the annual number of articles, countries (or regions) and institutions, authors and cited authors, journals and cited journals, and keywords were extensively analyzed to reveal the current research hot-spots and trends in this field.

Based on an analysis of countries (or regions) and institutions, the USA and China were the two most documented countries, but there was a lack of international cooperation between them. More international cooperation is needed for China (0.00 centrality) to jointly promote development in this field. England, Australia, and Canada had high centrality and publications, suggesting that these countries might play an essential role in bridging research. At the same time, most of the top ten institutions with the most centrality and publications are from China and the USA, but these institutions basically cooperate with other institutions in their countries. Therefore, from the perspective of cooperation networks, we hope that there will be more cooperation between different countries.

Through the analysis of the author cooperation network, we found that the top ten authors mainly constituted three cooperative networks. In the cooperation between the two authors in the deep learning field, VALENTINA PEDOIA (University of California System) and Sharmila MAJUMDAR (University of California System), the most cited literature showed that the improved U-net can automatically segment cartilage and meniscus from MRI (15). PAUL H YI (Johns Hopkins University, USA) and JAN FRITZ (New York University, USA) have reported that deep learning can significantly address the identification of implants after knee replacement surgery and help with subsequent revision surgeries (16). JARET M KARNUTA (Cleveland Clinic Foundation, USA), PREM N RAMKUMAR (Cleveland Clinic Foundation, USA) and HEATHER S HAEBERLE (Cleveland Clinic Foundation, USA) summarized some application of artificial intelligence in orthopedics (17). However, it is worth noting that in the entire network of cooperative map, none of the authors had a centrality <0.1, indicating a lack of international cooperation among these authors. The entire network presented a very weak partnership.

As to the analysis of a network of cited authors, the most frequently cited author-LeCun Y believed that deep learning is good at processing high-dimensional data, and can accurately identify and classify targets in image recognition tasks (18). Similarly, Litjens G et al. with the most centrality in the top ten co-cited authors published “A survey on deep learning in medical image analysis (19),” which plays a key role in the field.

According to the journals and co-cited journals in Table 4. The journal Skeletal Radiology had the most publications. The average impact factor of the top ten journals in 2020 was 4.086, and the impact factor of Medical Image Analysis was the highest (8.545). Furthermore, seventy percent of journals had an impact factor over three, and fifty percent of journals belonged to Q1 (JCR). The journal Nature had the highest impact factor in the cited journals. In addition to the cited journals that cannot obtain impact factor and JCR partitions, seventy-five percent of the cited journals belonged to Q1. These data suggested that this area has attracted academic attention from high-scoring journals.

Among top ten co-cited references, these references are usually the basis of relevant knowledge fields and play an important role in knowledge structure. For three proceedings papers, Ronneberger O, He KM, and Huang G built new models for deep learning in 2015, 2016, and 2017, respectively, which strengthened the ability of deep learning to process images. These models are deep residual learning, densely connected convolutional networks, and U-net (20–22). Article entitled “Deep learning” by LeCun Y et al. indicated that deep learning can precisely solve tasks including image recognition and speech recognition (18). Therefore, these articles laid the foundation of this field. “A survey on deep learning in medical image analysis” by Litjens G et al. summarized the research on the application of deep learning in various medical fields before Feb 1, 2017 in details, and these researches were basically published in 2016 and early 2017. We found that these studies on the application of deep learning in musculoskeletal disease were basically belong to proceedings papers (19). Today, the trend of publication in the journals is increasing year by year (Figure 1), suggesting that research in this field will still be a fertile area in the next few years. Among the top ten most frequently cited reference, a study by Olczak J et al. was the first article about deep learning application in fracture, and the performance of deep learning was like that of senior orthopedic surgeons (23).

Keywords are the cores of a paper that reflecting the concerns of relevant field. By analyzing the high-frequency keywords and strong-burst keywords, we can explore the hot-spots and research trends. The main research hotspots and research trends are as follows:

### For hot keyword-fracture

After the first report of deep learning in fracture by Olczak et al. (23), the number of studies concerning this field increased with years.

For upper extremity fracture, deep learning is commonly applied to distal radius fracture and humeral fracture (24–26). Gan et al. applied two deep learning models to identify distal radius fracture (24). They trained a Faster R-CNN with the ability to automatically annotate the regions of interest (ROIs), and then another CNN model for diagnostic was trained with images that were annotated by Faster R-CNN Model; The average intersection of the union (IOU) value of Faster R-CNN was 0.87, and the AUC of inception-v4 was 0.96. However, their data consisted only of anterior-posterior (AP) image, whereas images in both the AP and lateral directions were often clinically required, and there was no adequate amount of data from multiple centers. Recently, Suzuki et al. addressed these two issues, and the results showed that the CNN model had better accuracy than orthopedic surgeons (25). In addition to identification of fractures, deep learning can also directly classify fracture types. Chung et al. shown that CNN was superior to orthopedic surgeons in the classification of proximal humeral three- and four-part fracture, with an AUC exceeding 90%, while both fractures are easily misjudged in clinical work (27). Besides, Langerhuizen et al. tried to identify scaphoid bone fracture, a common fracture of the wrist, by using a small data set and found that deep learning performed inferior than surgeons in 2020 (28). However, Yoon et al. later established two model, namely the Apparent Fracture Model and the Occult Fracture Model, which excelled in identifying obvious and potential scaphoid fractures, respectively. Their research used more datasets and escalated deep learning algorithms, and both models showed higher sensitivity and specificity than that of Langerhuizen et al. (29).

In lower limb, hip fractures attracted much attention. Cheng et al. proved that deep learning model can automatically identify femoral neck fracture and trochanteric fracture through pelvic X-rays, with 98% sensitivity and 91% accuracy (30). Mutasa et al. and Bae et al. designed deep learning models for identifying garden-type femoral neck fractures on hip or pelvic X-rays, respectively (31, 32). Similarly, Urakawa et al. reported that deep learning also outperformed clinicians in identifying intertrochanteric fractures (33). In addition, Badgeley et al. incorporated patient characteristics into the deep learning model to predict hip fractures (34). Zdolsek et al. also identified atypical femoral fractures from normal femoral shaft fractures on conventional X-rays by deep learning models, and the ResNet had the best performance (35). Ankle fractures are considered as one of the most common fractures in clinical practice. Currently, classifying ankle fractures using the AO Foundation/Orthopedic Trauma Association (AO/OTA) system is not easy to grasp. The ResNet network trained by Olczak et al. can classify ankle fractures based on the AO/OTA system, with an AUC of 0.90 (36). In addition to these common lower extremity fractures, deep learning can also assist in diagnosing and identifying calcaneus fractures by accurately evaluating Bohler's angle (BA) and critical angle of Gissane (CAG) on X-rays (37).

Moreover, studies showed that deep learning can also help to reveal vertebrae fracture as well. Murata et al. accurately identified vertebral fractures on plain thoracolumbar radiography (38). Usually, compression fractures of the vertebrae are associated with osteoporosis. Improving the recognition of compression fractures is also the direction of the current solution. Now, deep learning can identify vertebral compression fractures on radiography and can help distinguish between fresh and old compression fractures, solving the challenge of identifying fresh compression fractures on radiography (39). Similarly, on MRI images, deep learning has also reached the level of specialists in identifying fresh compressed bones (40). More recently, a study by Suri et al. also adopted deep learning to accurately segment and identify the vertebral body and intervertebral discs on MRI, CT, and X-rays, which can offer substantial help in the clinical practice concerning spine-related diseases (41).

### For hot keyword-osteoarthritis

Osteoarthritis is the most common musculoskeletal disorder, which mainly affects the hip and knee joints with large weight bearings, especially the knee joint. Initially, a proceedings paper showed the use of CNN to analyze Kellgren & Lawrence (K&L) grades based on knee radiographs (42). Then, Tiulpin et al. incorporated additional disease-related features (such as the joint space) into the deep learning model and ultimately achieved better result (43). In addition, a deep learning model developed by Tiulpin et al. can automatically assess Osteoarthritis Research Society International (OARSI) and K&L grading (44). Leung et al. demonstrated the possibility of deep learning in predicting total knee replacement in patients with osteoarthritis. In addition to routine X-rays, current research suggests that deep learning can enable MRI to become an effective tool in osteoarthritis recognition and clinical application by significantly reducing the acquisition time and automatically precise segmentation (15, 45).

### For the burst keywords-risk factor, identification, localization, and surgery

Deep learning is often applied to the classification and diagnosis of diseases. However, with the development of deep learning, this technique will also be applied to perioperative management of orthopedic surgery. Nowadays, deep learning is being gradually explored in the study of orthopedic surgery. Since the visual field in arthroscopic surgery is often affected by different angles of the joint, which requires repositioning in different visual fields, automatic localization under the arthroscopic field of view is urgently needed. Recently, Banach A et al. collected arthroscopic videos and performed deep learning on video sequences at four different knee angles, and the results showed that deep learning performed well in arthroscopic field of view (46). Besides, Sarin JK et al. argued that deep learning can detect joint degeneration and help determine the boundaries between diseased cartilage and normal cartilage in arthroscopic surgery (47). In addition, Seibold M et al. combined acoustic sensing and deep learning to help reduce complications such as soft tissue damage caused by excessive bone drilling in surgery (48). These studies demonstrate the potential of deep learning in assisting orthopedic surgery. For the postoperative aspect of orthopedic surgery, the prediction of postoperative complications is important. Zhu WB et al. used deep learning to infer the possibility of bone necrosis after femoral neck fracture internal fixation from postoperative X-rays, which can help clinicians make timely treatment (49).

### For the timeline viewer of keywords

From the timeline viewer of keywords, we found that the study of fractures and osteoarthritis gradually began to study in-depth from 2016 to 2019. We also paid special attention to the direction of gradual in-depth research after 2019, such as osteoporosis, and bone tumors (osteosarcoma). Osteoporosis often occurs in older or postmenopausal women, and is usually detected because of fractures, thereby screening for osteoporosis may help prevent osteoporotic fractures in many patients. Deep learning can help predict osteoporosis and the possible fracture risk (50, 51). Although dual energy X-ray absorptiometry is the current gold standard for osteoporosis diagnosis, the lack of universality limits its clinical application. Accordingly, Loffler et al. used conventional CT combined with deep learning to predict osteoporosis (52). In addition, the present studies on bone tumor rely on radiographic imaging and histopathology. Deep learning can help improve the segmentation of bone tumor in imaging (53), and can also be used in histopathology to quickly distinguish between normal tumor areas and necrotic tumor areas, thereby assisting to evaluate response to neojuvant chemotherapy (54, 55). Therefore, the use of deep learning based on conventional imaging and histopathology to accurately screen osteoporosis, identify and classify bone tumors may become future research directions.

Several limitations remain in this study. First, as the literature in the Web of Science Core Collection is constantly updated, and there is currently no uniform regulation on keywords related to the literature, the results of this study may differ from the actual number of documents included. Second, deep learning first appeared in seminars or conferences, and then gradually appeared in journals in the form of article. Since we consider that article has more systematic research, so it is strictly regulated in literature inclusion, which may introduce certain bias to this study. However, it is believed that literature-based visual analysis has undoubtedly laid a foundation for investigators to quickly understand the research hot-spots and development trends of deep learning applied in orthopedics.

## Conclusion

In conclusion, according to the annual publication curve, the research on the application of deep learning in orthopedics has developed rapidly, and there is a good research prospect. Accordingly, researchers in the field of deep learning and orthopedics may benefit this study. The USA is the country with the largest amounts of articles in this field. University of California San Francisco, the institution with the largest amounts of articles, is also from the USA. However, cooperation between different countries is markedly insufficient. The application of deep learning in orthopedic research has been published in journals of various discipline categories, and multidisciplinary communication conforms to the mainstream of today's world. The current research hot-spots mainly focused on the classification and diagnosis of orthopedic diseases that rely heavily on medical images, such as fractures and osteoarthritis. The application of deep learning to reduce intraoperative risk, predict postoperative risk, screen osteoporosis, and identify, classify, and segment bone tumors may be at the fore-front of the future. At the same time, with the rapid development of deep learning applications in orthopedics, researches in this field will continue to evolve toward multicentric data and more perfect deep learning algorithms.

## Data availability statement

The original contributions presented in the study are included in the article/supplementary material, further inquiries can be directed to the corresponding author.

## Author contributions

CF: investigation, data analysis and visualization, and writing—original draft preparation. XZ, HW, YH, and ZL: investigation and editing. CT: conceptualization, supervision, and writing and revision. All authors have read and agreed to the published version of the manuscript.

## Funding

This work was supported by grant from the National Natural Foundation of China (81902745), Hunan Provincial Natural Science Foundation of China (2022JJ30843), the Science and Technology Development Fund Guided by Central Government (2021Szvup169), Hunan Provincial Administration of Traditional Chinese Medicine Project (No. D2022117), and Clinical Research Center for Medical Imaging in Hunan Province (2020SK4001).

## Conflict of interest

The authors declare that the research was conducted in the absence of any commercial or financial relationships that could be construed as a potential conflict of interest.

## Publisher's note

All claims expressed in this article are solely those of the authors and do not necessarily represent those of their affiliated organizations, or those of the publisher, the editors and the reviewers. Any product that may be evaluated in this article, or claim that may be made by its manufacturer, is not guaranteed or endorsed by the publisher.
